# Eumelanin Coating of Silica Aerogel by Supercritical Carbon Dioxide Deposition of a 5,6-Dihydroxyindole Thin Film

**DOI:** 10.3390/ma11091494

**Published:** 2018-08-21

**Authors:** Giuseppe Caputo, Irene Bonadies, Ludovico Migliaccio, Maria Federica Caso, Alessandro Pezzella

**Affiliations:** 1 Dipartimento Dell’Innovazione Industriale e Digitale-Ingegneria Chimica, Gestionale, Meccanica e Informatica, Università di Palermo, 90133 Palermo, Italy; giuseppe.caputo01@unipa.it; 2 Institute for Polymers, Composites and Biomaterials (IPCB), CNR, Via Campi Flegrei 34, I-80078 Pozzuoli (Na), Italy; irene.bonadies@ipcb.cnr.it; 3 Department of Chemical Sciences, University of Naples “Federico II” Via Cintia 4, I-80126 Naples, Italy; ludovico.migliaccio@unina.it; 4 Nanofaber Spin-Off at Italian National Agency for New Technologies, Energy and Sustainable Economic Development (ENEA), Casaccia Research Centre, Via Anguillarese 301, 00123 Rome, Italy; maria.federica.caso@gmail.com

**Keywords:** eumelanins, silica aerogel, supercritical CO_2_

## Abstract

Eumelanin integration in silica aerogel (SA) was achieved via supercritical adsorption of 5,6-dyhydroxyindole (DHI) from CO_2_. Notably, after the supercritical treatment, DHI evolved towards spontaneous polymerization, which resulted in uniform pigment development over the SA. The new material was characterized for its morphological and physicochemical properties, disclosing the formation of a eumelanin-like coating, as confirmed by UV–vis and electron paramagnetic resonance (EPR) spectroscopy.

## 1. Introduction

Eumelanin micro-and nanocoatings are emerging as a widespread tool for the fabrication of advanced multifunctional bio-interfaces [1,2]. Eumelanin biopolymers are formed by the oxidative polymerization of the amino acid tyrosine [3], and their use within interfaces to provide substrates with the enhanced chemical-physical properties of the poly-hydroxyindole backbone and, at the same time, with adhesion [4], bio-compatibility [5], electrical conductivity [6], and UV-vis absorption ability [2] is under exploitation.

Aerogels [7] are highly cross-linked, three-dimensional, highly porous solids usually featuring a random interconnection of nano-networks of particles and wires associated with noteworthy properties, including low density, high porosity, low dielectric constant, high specific surface area, ultralow thermal conductivity [8]. Among the most exploited protocols for aerogel fabrication, supercritical point drying (SCD) is the most versatile, as it allows to employ two basic strategies, i.e., high-temperature (HT) and low-temperature (LT) drying [9], the latter often based on the use of the non-flammable and “green” carbon dioxide. In recent years, because of their distinctive properties, aerogels have received increasing attention for their potential application in drug delivery systems, solid-phase catalysis, thermal and acoustic insulation, adsorbent units fabrication, and as templates for active coating [8,10,11,12].

Silica aerogels are highly investigated in view of a wide variety of applications [13]; however, they suffer from some mechanical weakness [14,15] which appears to even increase in water media [16].

Several attempts have been made in order to improve silica aerogel properties [11], including the integration of carbon materials [17,18], which appears one of the most studied and versatile approaches [19]. In this scenario, a recent study focused on the integration of a silica aerogel and graphene oxide, which improved the mechanical strength as a result of the strong silica–graphene oxide interaction [20].

In order to investigate the possibility to pair the carbon-driven improvement of silica aerogel properties with the integration of a biocompatible aromatic polymer, we devised an ad-hoc adsorption isotherm protocol involving the addition of 5,6-dihydoxyindoled (DHI) to a commercial silica aerogel and resulting in a eumelanin-coated hybrid (EuSiGel).

## 2. Experimental

### 2.1. Materials

Hydrophilic silica aerogel (SA) in form of monolithic blocks was purchased from Merketech Int. (Port Townsend, WA, USA). The nominal density was 0.1 g/cm^3^, the surface area was 800 m^2^/g, and the mean pore size was about 20 nm. Coating experiments were performed on nearly cubic blocks of 1 cm obtained from SA monoliths. CO_2_ research-grade 4.8 was purchased from Air Liquide (Italy).

5,6-Dihydroxyindole (DHI) was prepared following a reported procedure [4]. Other commercially available chemicals were used without further purification.

### 2.2. Apparatus

The main part of the apparatus (laboratory-scale) was an autoclave, featuring a stainless steel cylinder (internal volume of 50 mL), capped on the bottom and on the top with two plates. The maximum operating pressure was 200 bar. Mixing was provided by a magnetic stir bar placed on the bottom of the autoclave. The autoclave was heated by ceramic heaters, whose thermal control was guaranteed by a PID controller. The temperature inside the cylinder was measured by a J-type thermocouple with an accuracy of ± 0.1 °C; a digital gauge manometer allowed pressure recording. At the exit of the autoclave, a rotameter was used to measure CO_2_ flow rate. Depressurization was obtained through a battery of two micrometering valves connected in series. Carbon dioxide was delivered to the autoclave by a diaphragm piston pump (Dosapro Milton Roy, Pont-Saint-Pierre France) with a maximum working pressure of 300 bar. A cooling bath connected to the pump head allowed the cooling of CO_2_ before compression. After optimization, working temperature and pressure were established as 40 °C and 120 bar.

### 2.3. Adsorption Experiments

Adsorption isotherm experiments were carried out using a static method [21]. The autoclave was loaded with SA (about 0.35 g, total weigh of several small SA samples introduced) cubic monoliths placed in the bottom of the autoclave and wrapped in filter paper to avoid their contact with solid DHI. A weighed amount of DHI (0.1 g, in excess with respect to its solubility in CO2 in the experimental conditions) was placed in a top-opened vessel to allow contact with CO_2_ and mounted on an axial bar fixed to the top cap of the autoclave. The autoclave was closed, heated to the desired temperature, and CO_2_ was slowly added to the system at a constant flow rate. The amount of CO_2_ in the autoclave was calculated from the density data. When the working pressure was reached, the system was allowed to stand for a fixed time. Then, CO_2_ was constantly vented with a pressure drop of about 0.5–1 bar/min. After the vessel was cooled, the impregnated SA was removed and weighed. The amount of adsorbed compound was determined by the weight change of the SA, using an analytical balance. Because CO_2_ is also adsorbed on SA under pressure and slowly released at ambient conditions, the final weight of the loaded SA was determined after all CO_2_ had been released from SA. This condition was achieved when the weight of the sample became stable. The amount of DHI that can be adsorbed on SA depends both on the kinetics and on the thermodynamics of adsorption from the gas phase.

### 2.4. Analytical Methods

UV–vis optical absorption analysis of layers deposited on appropriate substrates was carried out by using a Perkin-Elmer Lambda 900 spectrophotometer (Waltham, MA, USA).

Electron paramagnetic resonance (EPR) measures were taken with an X-band (9 GHz) Bruker Elexys E-500 spectrometer (Bruker, Rheinstetten, Germany), featuring a super-high sensitivity probe head. The solid samples were introduced and sealed in glass capillaries which, in turn, were coaxially inserted in a standard 4 mm quartz sample tube. The measurements were performed at standard conditions.

Main settings adopted: sweep width, 100 G; resolution, 1024 points; modulation frequency, 100 kHz; modulation amplitude, 1.0 G. The amplitude of the field modulation was verified to be low enough to avoid detectable signal over modulation. Up to 64 scans were accumulated to improve the signal-to-noise ratio. In power saturation experiments, the microwave power was gradually raised from 0.02 to 160 mW. The g-factor value was evaluated by means of an internal standard (Mg/MnO).

Aerogel surfaces were evaluated by a field-emission scanning electron microscopy (FESEM, QUANTA200, FEI, Eindhoven, The Netherlands) at an accelerating voltage of 30 kV after gold–palladium sputter coating. The elemental composition measurements were carried out with an energy dispersive X-ray spectroscopy (EDS) detector for elemental analysis on samples without the metallic coating.

The thermal stability of the samples was evaluated by thermogravimetric analysis (TGA) performed with a Perkin-Elmer Pyris 1. The samples were heated in an oxidative environment (air, 30 mL/min) from 50 °C to 800 °C at a rate of 10 °C/min.

## 3. Results and Discussion

The adsorption of DHI on SA was carried out at 40 °C and 120 bar. In these conditions, CO_2_ density is 0.761 g/cm^3^, which is a reasonable value to ensure dissolution of DHI. The adsorption process was carried out for a period of 24 h to guarantee that equilibrium was reached. Moreover, the adsorption experiments were repeated three times with satisfactory reproducibility. After opening of the autoclave, the presence of DHI into the SA structure was easily detected by the change of colour of SA that assumed a brown colour. Although SA is fragile, the monoliths were not broken during loading and venting of CO_2_ from the autoclave. The amount of DHI adsorbed on SA, measured by the weight change of the samples before and after the experiment, was 2.3% with respect to the initial weight of SA.

The weight percent data were confirmed by thermogravimetric analysis, measuring the mass loss of the samples upon heating from room temperature up to 800 °C in an oxidative environment. In these conditions, both SA and EuSiGel experienced a very limited weight loss of few percentage points.

**Figure 1:**
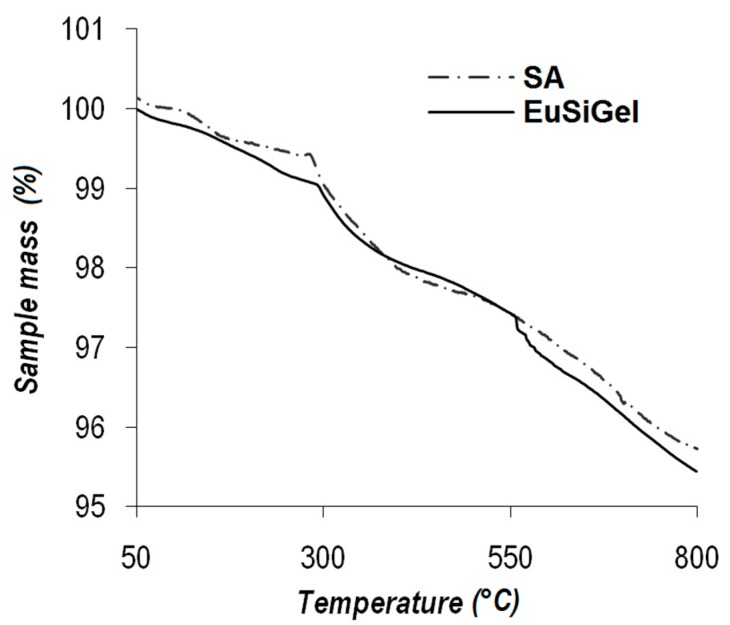
Thermogravimetric analysis of the parent silica aerogel (SA) and the eumelanin-coated hybrid (EuSiGel) in an oxidative environment (air).

The TGA curves (Figure 1) of SA and EuSiGel exhibited a quite similar profile up to a loss difference at 550 °C, corresponding to the significant oxidation of the eumelanin component [18], which was likely due to the decomposition of labile oxygen-bearing functional groups. The silica component produced some increase of the eumelanin degradation temperature [18], probably because of the insulator effect of the aerogel. Both samples exhibited a comparable weight loss below 800 °C, as reported for other glassy materials [22,23].

Notably, even if the fabrication conditions of the EuSiGel involved oxygen-free atmosphere, the combination of pressure, temperature, and the silica surface induced DHI polymerization and the formation of the typical eumelanin pigment within the SA scaffold. Although photo- as well as thermo-induced processes may not be excluded, it may be speculated that silica acidic groups at the aerogel nanostructured surface can promote DHI polymerization [24,25]. Although the term eumelanin designates different pigments obtained from related precursors [1], DHI stands among the most investigated sources of dark melanin pigments, and evidence in the literature indicates a strong matching between the pigment from DHI oxidative polymerization and natural eumelanins [1]. On this base, the term eumelanin will be used in the following discussion to denote the investigated material.

**Figure 2:**
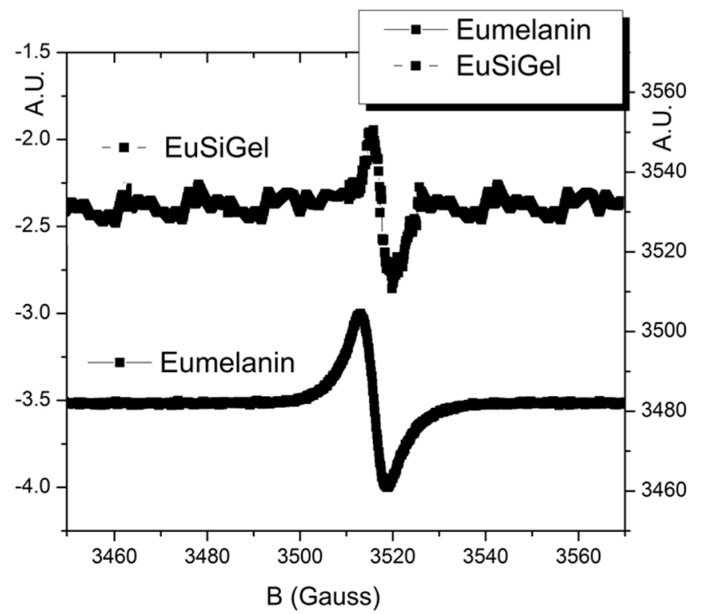
Electron Paramagnetic Resonance (EPR) spectra of the EuSiGel (top line) and reference eumelanin (bottom line).

The effective eumelanin formation was checked by EPR and UV–vis spectroscopy in order to check the typical eumelanin features (Figure 2). Indeed, the EPR spectrum, although highly noisy, showed a line shape virtually superimposable to the one of the reference eumelanin sample, featuring a single, roughly symmetric signal at a g value in the range of ~2.0030–2.0040 and a ΔB(G) of 5.1 ± 0.5. This value is associated with carbon-centered radicals formed during DHI polymerization [26] and is a typical signature of the eumelanin pigment [2].

**Figure 3:**
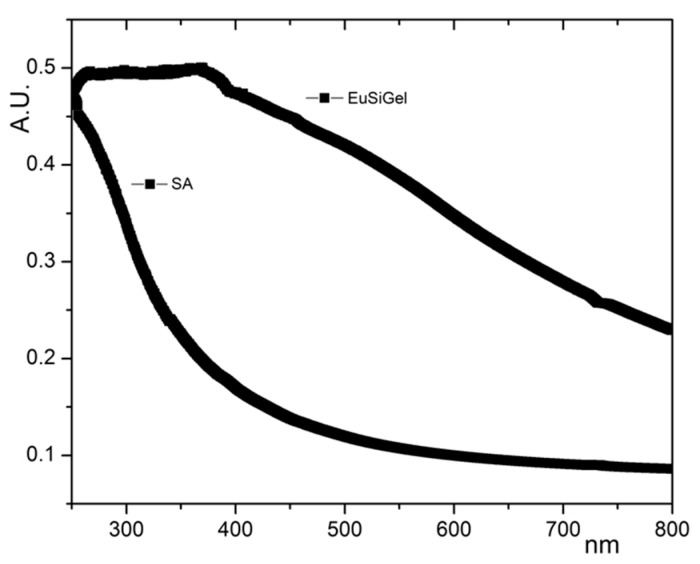
UV–vis absorbance spectra of EuSiGel and reference SA.

Coherent evidence was provided by the UV–vis spectra (Figure 3) of the EuSiGel and SA. The SA spectrum lacked significant components in the visible region, whereas the EuSiGel spectrum displayed the typical feature of eumelanin, i.e., a significant absorption in the visible region. This last feature, distinctive of the eumelanin pigment, is ascribed to the polyindole backbone and is a proof of the presence of an extended conjugated system within the scaffold.

**Figure 4:**
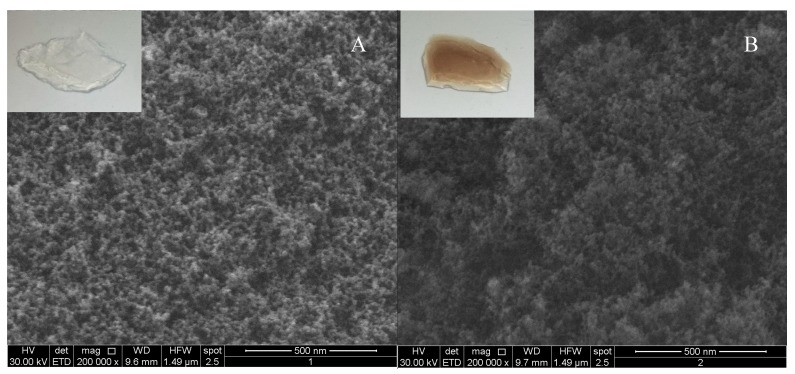
SEM images of (**A**) reference SA and (**B**) EuSiGel. Pictures of the corresponding samples are also reported (upper-left corner).

A morphology inspection, carried out by SEM microscopy, proved the highly homogeneous loading of eumelanin within the EuSiGel. Figure 4 reports SEM images of the silica aerogel and EuSiGel and the pictures of the corresponding samples, disclosing the complete retain of the silica surface pattern after eumelanin coating. This result demonstrates the effectiveness of DHI delivery to the silica aerogel under supercritical condition to obtain a eumelanin-containing aerogel without losing the original structural and morphological characters of the aerogel.

## 4. Conclusions

The integration of eumelanin pigment and silica aerogel was reported, describing a DHI-based route to the formation of a eumelanin–aerogel composite featuring high-quality morphology (SEM evidence) and the eumelanin physicochemical signature, i.e., UV–vis broadband monotonic absorption. The composite was obtained by easy impregnation of silica aerogel with DHI under supercritical CO_2_, with complete retain of the morphological and structural features of silica aerogel.

The results herein reported expand the scope of eumelanin coatings to low-density high-surface materials, providing a clear-cut route to silica aerogel functionalization via a biocompatible eumelanin interface.
